# An integrated metagenomic, metabolomic and transcriptomic survey of *Populus* across genotypes and environments

**DOI:** 10.1038/s41597-024-03069-7

**Published:** 2024-04-05

**Authors:** Christopher Schadt, Stanton Martin, Alyssa Carrell, Allison Fortner, Dan Hopp, Dan Jacobson, Dawn Klingeman, Brandon Kristy, Jana Phillips, Bryan Piatkowski, Mark A. Miller, Montana Smith, Sujay Patil, Mark Flynn, Shane Canon, Alicia Clum, Christopher J. Mungall, Christa Pennacchio, Benjamin Bowen, Katherine Louie, Trent Northen, Emiley A. Eloe-Fadrosh, Melanie A. Mayes, Wellington Muchero, David J. Weston, Julie Mitchell, Mitchel Doktycz

**Affiliations:** 1 Biosciences Division, Oak Ridge National Laboratory, Oak Ridge, TN USA; 2 Environmental Sciences Division, Oak Ridge National Laboratory, Oak Ridge, TN USA; 3 Environmental Genomics and Systems Biology Division, Lawrence Berkeley National Laboratory, Berkeley, CA USA; 4 Pacific Northwest National Laboratory, Richland, WA 99354 USA; 5 Bioscience Division, Los Alamos National Laboratory, Los Alamos, NM 87545 USA; 6 DOE Joint Genome Institute, Lawrence Berkeley National Laboratory, Berkeley, CA USA; 7 Present Address: Division of Computational Biology, Mayo Clinic, Rochester, MN 55905 USA

**Keywords:** Plant ecology, Metagenomics, Microbial ecology

## Abstract

Bridging molecular information to ecosystem-level processes would provide the capacity to understand system vulnerability and, potentially, a means for assessing ecosystem health. Here, we present an integrated dataset containing environmental and metagenomic information from plant-associated microbial communities, plant transcriptomics, plant and soil metabolomics, and soil chemistry and activity characterization measurements derived from the model tree species *Populus trichocarpa*. Soil, rhizosphere, root endosphere, and leaf samples were collected from 27 different *P. trichocarpa* genotypes grown in two different environments leading to an integrated dataset of 318 metagenomes, 98 plant transcriptomes, and 314 metabolomic profiles that are supported by diverse soil measurements. This expansive dataset will provide insights into causal linkages that relate genomic features and molecular level events to system-level properties and their environmental influences.

## Background & Summary

Being the primary means of atmospheric CO_2_ fixation in terrestrial ecosystems, plants provide a large potential sink for this greenhouse gas^1^. Plants can enhance carbon uptake under elevated CO_2_ concentrations, have capacity to store carbon in their root systems, and can further transfer carbon to long residence time pools in the soil. However, ecosystem responses to climate change drivers are complex, as studies have demonstrated that soil nutrient limitations can reduce growth and alter plant carbon allocation below ground, and therefore limit carbon storage^2^. Indeed, effective modeling of the carbon cycle also requires consideration of nitrogen cycle influences^3^. Further influencing these host and edaphic factors are microbial interactions, as numerous studies confirm that microbial interactions affect plant carbon gain and allocation, nutrient acquisition, plant biomass yield, and soil carbon and nitrogen cycling^4^. The inability to untangle these biological, physical, and environmental variables is a serious impediment to efforts seeking to understand and reduce climate change effects on managed and natural ecosystems.

Plants have variable affinities and efficiencies for N uptake across genetically diverse populations^5^, that result in variable leaf and root C/N ratios and photosynthesis rates^6^, and ultimately carbon storage potential. Plants also produce chemical signals and allelopathic compounds to alter the functions of the plant-associated microbiome. One such mechanism is through production of compounds that cause biological nitrification inhibition [BNI] and biological denitrification inhibition [BDI] effects on ammonia oxidizing and denitrifying bacteria and archaea, respectively, that in turn influence N availability, N runoff and aquatic organisms, as well as production of the greenhouse gas nitrous oxide^7–10^.

**Fig. 1:**
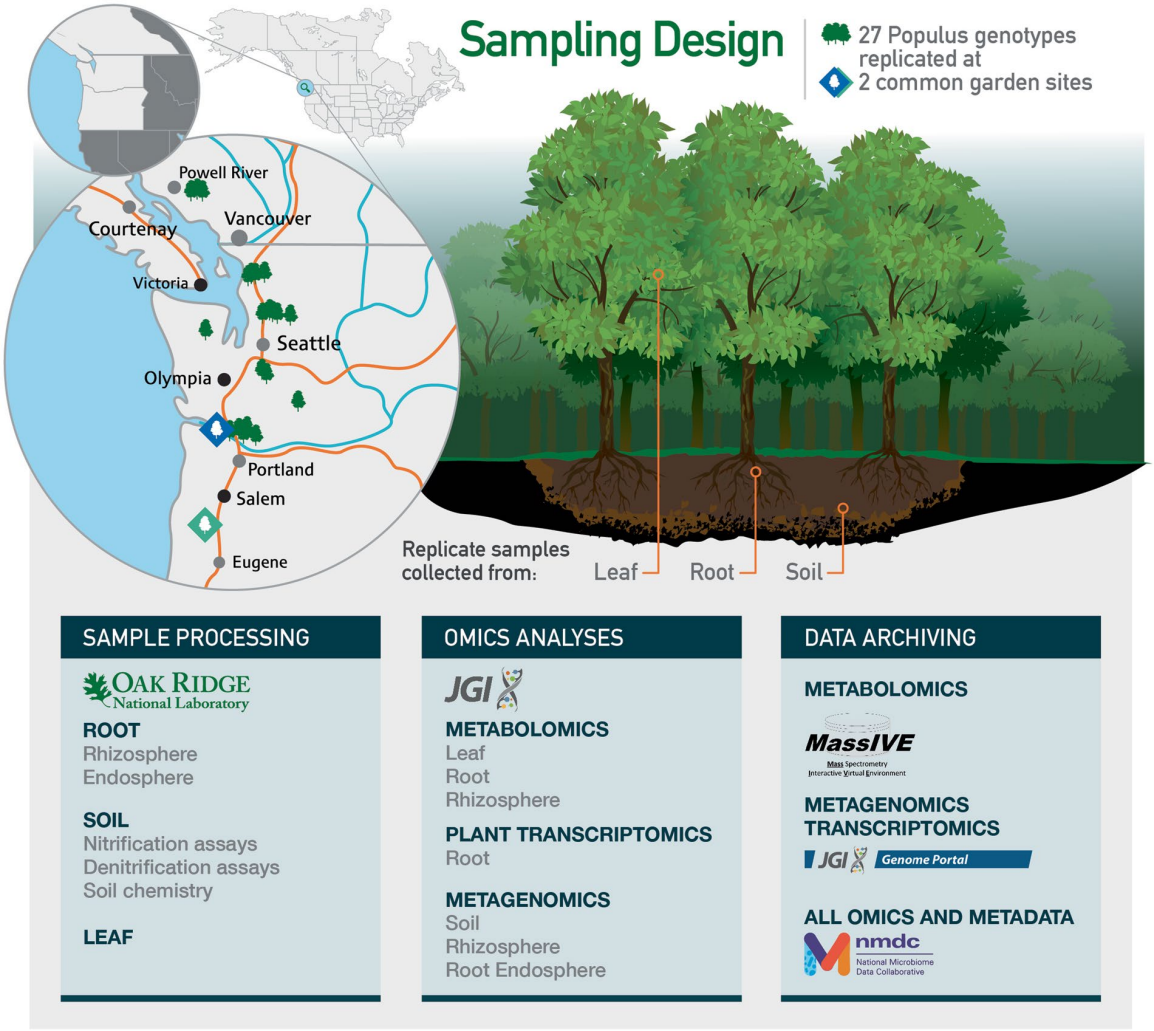
Overview of sample and information flow. 27 *Populus trichocarpa* genotypes, growing in common gardens located in Clatskanie, OR (blue diamond) and Corvallis, OR (green diamond) were sampled. These natural variants were obtained from locations throughout the Pacific Northwest (green tree icons). Replicate leaf, root, and soil samples were collected. Root samples were prepared for transcriptomics experiments and further divided into rhizosphere and endosphere samples for metagenomics analyses. Soil samples collected near the tree were prepared for chemical analyses and for metagenomics. Metabolomics experiments were carried out on leaf, root, and rhizosphere materials and archived in the MassIVE database. Metagenomics and transcriptomics data are accessible at the JGI Genome Portal. Metadata, and links to all omics data, are accessible at the NMDC.

Integrated ‘omics approaches can reveal how plant related molecular and cellular events are connected to ecosystem processes^11^. As a model for such important studies, *Populus* species are ideal as they are transcontinental in their natural distributions, ecologically and commercially important, currently used in the pulp and paper industry, and have demonstrated potential as bioenergy feedstocks^12^. In this Data Descriptor we present an integrated dataset which contains metagenomics data from plant-associated microbial communities, plant transcriptomics, plant and soil metabolomics, and soil chemical and activity characterization measurements derived from different *Populus trichocarpa* genotypes grown in two different common garden environments (Fig. 1). The sampling design involved collection of soil, rhizosphere, root endosphere, and leaf samples from up to three replicates of 27 different *P. trichocarpa* genotypes grown at each of two common gardens. In total, the final curated dataset includes 318 metagenomic samples prepared from soil, rhizosphere, and the root endosphere, along with 98 plant root transcriptomics profiles. These sequencing datasets are complemented with 314 metabolomics measurements derived from root, rhizosphere soil, and leaf samples. Supplementing this dataset are soil measurements including N pools (total N, NH_4_-N and NO_3_-N), soil nitrification and denitrification potential assays, as well as previously published host genome sequences and SNPs for the genome-wide association study (GWAS) population^13^, and additional types of metadata.

This combined dataset will facilitate the use of multivariate statistical and machine learning approaches that have the potential to identify the causal linkages and mechanisms that relate molecular level events to ecosystem processes. These data, to the best of our knowledge, represent the most comprehensive, publicly available, fully integrated *Populus trichocarpa* multiomics dataset from field samples collected to date and should allow for understanding of how the genetic, phenotypic, and chemotypic diversity of *Populus trichocarpa* relate to ecosystem services such as coupled soil nitrogen and carbon cycling processes.

## Methods

### Field sampling and soil analyses

Candidate genotypes were sampled from each of two common gardens near Corvallis and Clatskanie OR in September 2020; *Populus trichocarpa* populations for which extensive prior GWAS data exist^13–15^. Both field sites were planted in 2009, however the Corvallis site is coppiced every two to three years and were thus functionally 3 years old at time of sampling. Most of the replicates in the Clatskanie site have never been coppiced (about one third were coppiced once in 2011) and thus were functionally 9–12 years old. The soils and climate of the two locations are quite different, with the plantations near Clatskanie occupying a diked former floodplain of the Columbia River, and the area experiences warm moist summers and cool wet winters. Inceptisols from the Wauna Series and Entisols from the Locoda Series silt loams were formed in recent silty alluvium^16^. The soils are deep and poorly drained and contain 10–35% clay and 60% silt. All soils are strongly acidic, with pH ranging from 4.6 to 5.4. The soils also exhibit redox mottling, suggestive of significant anaerobic and aerobic periods. Plantations near Corvallis in contrast are alluvium from the Williamette River valley and consist of deep Mollisols with texture of fine to gravely sandy loams. These soils are from the Newberg and Camas Series and experience warm dry summers with longer drought periods, cool moist winters, and lower overall precipitation compared to Clatskanie^16^. The Corvallis soils are excessively drained, contain 10% clay and 70% sand, and are moderately acidic to near neutral (pH 5.8 to 6.3).

Each sampled *P. trichocarpa* genotype was selected based on prior greenhouse and field data that showed them to have either significantly higher or lower concentrations of p-coumaric, ferulic, or alpha-linolenic acids compared to others in the GWAS population, as these compounds have been shown to influence nitrification and denitrification processes in other studies^17,18^. A summary of the selected genotypes and the relative levels of these originally measured metabolites are described in Table 1. The GWAS populations and common gardens from which these samples were taken have been described in prior publications^13–15^. In brief, the population includes three clonal replicates of each of 1100 natural variants of *P. trichocarpa* collected across the majority of its range from British Columbia through California that have been fully re-sequenced by the Joint Genome institute. The clonal replicates of each tree were planted in 2009 and laid out in three different blocks in both Clatskanie and Corvallis. Three replicate samples of 19 genotypes (57 trees) representing these categories were collected from the Corvallis location; however, in some cases all three replicate genotypes collected in Corvallis were not available in the Clatskanie location due to differential mortality between the sites. In these cases, genotypes with similar chemical phenotypes were selected as alternates in each category and sampled at Clatskanie (totaling 52 trees, representing 25 genotypes at Clatskanie). Thus, in total 27 genotypes were sampled across both sites. Within 2 meters of each selected tree, samples were collected for soil, rhizosphere, and root endosphere metagenomics analyses; a separate set was split in the field for rhizosphere, root, and leaf metabolomic analyses as well as root transcriptomic analyses. Each sample was frozen on dry ice in the field and shipped to the laboratory where they were stored at −80 °C until processing. Leaf sampling targeted the 3 to 5^th^ fully expanded leaves and those exposed to direct sunlight, however this was not always possible at the Clatskanie site due the size of the trees and closure of the canopy. Root and rhizosphere samples were obtained by carefully excavating and tracing roots attached to the base of each tree until fine roots were found in order to ensure they were directly associated with the selected tree genotype. Separate soil samples were also collected under each tree for analysis of physical and chemical properties, as well as nitrification and denitrification potential assays. These samples were placed on ice in the field and shipped to the laboratory where they were stored at 4 °C until processing. Soil chemical and physical characterization was performed at University of Georgia Agricultural & Environmental Services Laboratories and included elemental soil analyses, pH and salt concentrations, lime buffering capacity, soil moisture content, soil particle size analyses, nitrate and ammonium levels, and percent carbon and nitrogen. Soil particle size, as well as nitrification and denitrification potential assays, were performed at ORNL using protocols described previously^19–21^.

**Table 1:** Sampled genotypes were selected based on the predicted relationships between genotypes in rank distributions of prior chemical metabolite data from greenhouse and field studies performed on leaves.

Genotype ID	Corvallis Replicates	Clatskanie Replicates	alpha-linolenic acid	p-coumaric acid	ferulic acid
BESC-198	0	2	—	High	—
BESC-258	0	2	Low	—	—
BESC-285	0	2	—	—	Low
BESC-307	3	2	—	—	High
BESC-331	3	1	—	High	—
BESC-351	3	3	—	Low	—
BESC-360	3	2	High	—	—
BESC-388	3	3	—	Low	—
BESC-833	3	2	—	—	High
BESC-847	3	3	High	—	—
BESC-86	0	1	—	—	Low
BESC-866	3	2	High	—	High
BESC-904	3	2	High	High	—
GW-11047	0	2	—	High	—
GW-4579	3	3	Low	—	—
GW-9591	3	3	—	—	Low
SKWA-24-3	3	1	Low	—	—
SKWD-24-1	3	1	Low	—	Low
BESC-905	0	1	High	—	—
BESC-448	0	2	Low	—	—
BESC-845	3	3	—	High	—
BESC-133	3	3	—	Low	—
BESC-470	0	1	—	—	High
BESC-234	3	2	—	—	Low
BESC-13	3	3	—	High	—
GW-7986	3	0	—	Low	—
BESC-56	3	0	—	Low	—

### Preparation of samples for metagenomic, metabolomic and transcriptomic analyses

**Fig. 2:**
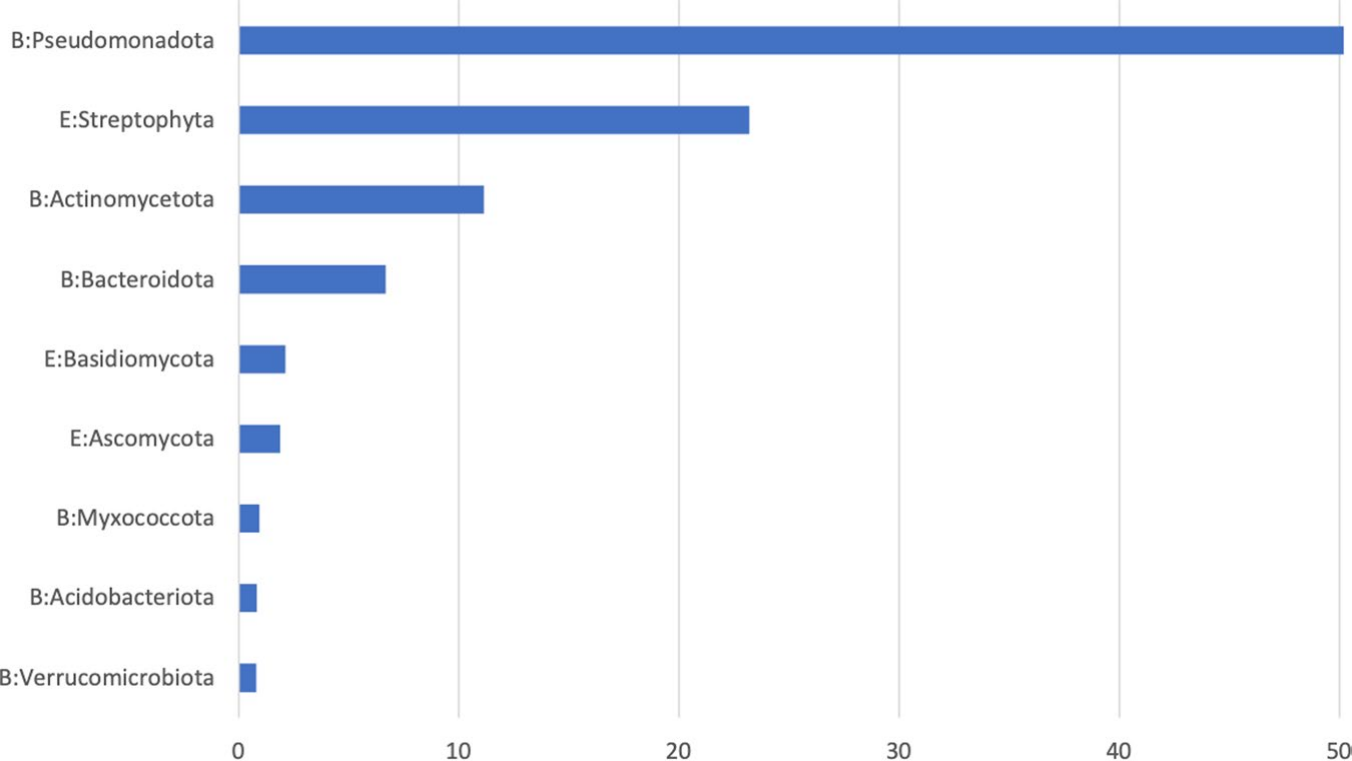
Phylogenetic origin of genes identified across all root endosphere metagenome hits in the assembled libraries were assessed at a >60% AA identity threshold in JGI Genome Portal. Streptophyta, the phylum containing plants and *Populus trichocarpa*, were found to comprise 23.2% of genes identified in the assemblies suggesting plant background contamination is minimized with these protocols. Only phyla comprising >0.5% of the hits across the entire assembled dataset are shown above. Together these phyla constitute >97% of the endosphere metagenomic dataset.

Soil, rhizosphere, and root endosphere samples for metagenomic studies were processed as previously described^22^ prior to extraction. Metagenomes were characterized using either standard True-Seq (soil and rhizosphere) or Nextera XT Low-Input protocols (root endosphere) in collaboration with the DOE Joint Genome Institute (JGI). A challenging aspect of metagenomic analysis of root endosphere microbiomes is the high background of plant genomic DNA that can dominate sequencing results. We previously developed a protocol to minimize this “host background contamination” however it required the use of a differential ultra-centrifugation protocols and required tens of grams of root material and resulted in only 10 s of nanograms of DNA^23^. Here, we used a new simplified centrifugation-based approach, which speeds the separation of host cells before extraction, to streamline and miniaturize the preparation of endosphere metagenomic samples. This procedure was modified from the method cited above. Briefly, in order to focus analyses on the most active roots^24^, fine roots <2 mm were subsampled from each collection, were frozen in liquid nitrogen, ground using a SPEX Geno/Grinder system, and then 0.5 grams of ground material was transferred into 2 mL screw cap tubes containing 0.3 grams of sterile 0.1 mm silica/zirconia beads. 1000 µL of sterile potassium phosphate buffer (10 mM, pH 6.5) was then added to each tube and disrupted further using a Retch MM400 Mixer Mill for 3 min at 30 Hz. Tubes were then centrifuged for 5 min at 500 g at room temperature and supernatant removed to a new 2.0 mL tube which was then centrifuged for 30 min at 12000 g at 4 °C. The supernatant was then discarded, and the pellet containing enriched microbial and depleted plant material was frozen at −20 °C until DNA extraction. DNA was extracted from these endosphere pellets, as well as for soil and rhizosphere samples, using standard Qiagen DNeasy Powersoil Kit protocols. Endosphere sample DNA was then amplified for metagenomic analyses using Nextera XT indexing and Low-Input protocols using the Illumina DNA prep kit with 8 bp Unique Dual indexes (UDI) at ORNL. Amplification cycles with the Nextera preps varied from 12 to 17 cycles, depending on the initial concentration of DNA in the sample. Samples were then shipped to the JGI on dry-ice for Illumina NextSeq sequencing. Initial results from these approaches (Fig. 2) show plant host contamination is minimized to on average around 23% of each metagenome. Unamplified rhizosphere and soil sample metagenomes were prepared directly from DNA extractions and shipped to JGI on dry-ice for sequencing using the standard TruSeq protocol.

Leaf, root, and rhizosphere soil samples for metabolomic and transcriptomic analyses were collected as separate samples in the field and processed as follows. Roots from each sample were placed in a ceramic or stainless-steel mortar, bathed in liquid nitrogen. Rhizosphere soil samples (consisting of soil originally adhering to the root samples collected from the same sample bag) was immediately subsampled and refrozen. To focus on the most biologically active portion of the root sample, roots <2 mm in diameter were quickly separated using tweezers from overall samples in the mortar, placed into a new tube on dry ice while processing, and then refrozen. Subsamples from all three metabolomic sample types (leaf, root, and rhizosphere) were then frozen in liquid nitrogen and ground using a SPEX Geno/Grinder system. The remainder of the root sample available after the metabolomics preps was then used for plant transcriptomic analyses. However, because the amount of material remaining in some cases was not enough for RNA extraction 18 (of 109) samples were omitted. Root transcriptomics samples were prepared for extraction by freezing fine roots in liquid nitrogen within a 50 mL grinding jar and grinding for 30 s with a steel ball at 30 Hz on a Retch MM400 Mixer Mill. Total RNA was then extracted 100 mg of ground material from each sample using a combined method that included CTAB lysis buffer and a Spectrum Total Plant RNA extraction kit (Sigma) as described previously^25^. RNA was then shipped to JGI on dry ice for cDNA preparation and sequencing.

Extractions for metabolomic analyses were then further prepared by JGI. For general non-targeted metabolomic analysis by liquid chromatography - mass spectrometry (LC-MS), polar and non-polar metabolites were extracted from rhizosphere soil, leaf, and ground root tissue using a methanol-based extraction. Here, leaf and root samples were first frozen and lyophilized dry (FreeZone 2.5 Plus, Labconco), then powderized by bead-beating using a FastPrep-24 5 G (MP Biomedicals) for 5 seconds (2x) using a 2 mm stainless steel bead. To extract leaf and root metabolites, 1 mL of 100% methanol was added to 300–400 mg powderized root tissue or 500–600 mg powderized leaf tissue in 2 mL Eppendorf tubes, briefly vortexed, sonicated 10 minutes in an iced water bath, centrifuged 5 minutes at 4000 rpm, then supernatant containing extracted metabolites were transferred to new 2 mL Eppendorf tubes and dried in a SpeedVac (SPD111V, Thermo Scientific). To extract soil metabolites, 4 mL of 100% methanol was added to ~500 mg soil samples in a 15 mL Falcon tube, briefly vortexed, sonicated 10 minutes in an ice water bath, then centrifuged 5 minutes at 4000 rpm. Supernatants were then syringe-filtered using 0.45 µm hydrophilic PVDF membranes (Pall), transferred to 5 mL Eppendorf tubes and dried in a SpeedVac. Dried metabolite extracts were stored at −80 °C until ready for LC-MS.

For quantitative LC-MS, dried sample extracts were resuspended in 100% methanol containing isotopically labeled internal standards (5–50 μM of ^13^C, ^15^N Cell Free Amino Acid Mixture, #767964, Sigma; 1 µg/mL 2-amino-3-bromo-5-methylbenzoic acid, ABMBA, #R435902, Sigma; 10 µg/mL ^13^C-trehalose, #TRE-002, Omicron; 10 µg/mL ^13^C-mannitol, ALD-030, Omicron; 2 µg/mL ^13^C-^15^N-uracil, CNLM-3917, CIL; 5.5 µg/mL ^15^N-inosine, NLM-4264, CIL; 4 µg/mL ^15^N-adenine, NLM-6924, CIL; 3 µg/mL ^15^N-hypoxanthine, NLM-8500, CIL; 5 µg/mL ^13^C-^15^N-cytosine, #294108, Sigma; 2.5 µg/mL ^13^C-^15^N-thymine, CNLM-6945, CIL; 1 µg/mL ^13^C linolenic acid, #CLM-8386, CIL; 1 µg/mL ^13^C-ferulic acid, #CLM-9260, CIL; 1 µg/mL ^13^C-p-coumaric acid, #CLM-10642, CIL; 1 µg/mL ^13^C-L-malic acid, #CLM-8065, CIL; 1 µg/mL ^13^C-citric acid, #CLM-9876, CIL; 1 µg/mL ^13^C-D-sucrose, #CLM-8091, CIL). Resuspension volumes were varied for each sample to normalize by initial biomass (200 µL per 150 mg for leaf and root, 200 µL per 500 mg for rhizosphere soil). Either 150 or 200 µL of resuspended sample volume were then centrifuge-filtered (0.22 μM hydrophilic PVDF membrane, #UFC30GV00, Millipore) and transferred to glass LC-MS vials.

Within the targeted identifications files, observations that exceed level 1 represent our highest level of confidence in that they match the fragmentation data, expected retention time, and have no confounding signals within our mass tolerance for the precursor m/z. Towards a larger scope of potential compound identifications, several observations within the targeted analysis match only the expected retention time and precursor m/z; while these are considered Level 1 identifications, they are far lower confidence than the aforementioned observations. The largest possible scope of potential compound identifications can be seen in the untargeted data. These matches are only when fragmentation data from our experiment agrees with fragmentation data from authentic standards. These identifications should be considered as putative identifications but nevertheless can be extremely useful to advance discovery due to their broad coverage of the metabolome.

### Metagenome sequencing and data processing

Metagenome sequencing was performed at the JGI under award number 507130 using their standardized approaches^26^. Briefly, paired-end sequencing was performed using the NovaSeq 6000 instrument from Illumina with an S4 type flow cell. Sequencing reads were then quality controlled for length (>50), quality (>Q30) and any adapter contamination removed using the JGI BBTools program suite^27^. This parsed readset was then assembled using metaSPAdes assembler version 3.15.2^26^ using default parameters. Further annotation and data processing was performed using the DOE-JGI Metagenome Annotation Pipeline (v.5.0)^28^. Summary statistics for the number of samples, the average depth of sequencing, and other functional information derived from the metagenomics analyses are described in Table 2.

**Table 2:** Summary of metagenomic sequencing dataset after final QC and assembly.

GWAS Common Garden Location	# of genotypes/individuals sampled	Sample type	# of metagenomes passing final QC	Avg number of assembled reads per metagenome	Avg gene count per metagenome	Avg number of genomes represented	Avg KEGG count
Corvallis, OR	19/57	Soil	57	50,560,875	1,743,651	108	175,436
		Rhizosphere	57	91,176,939	972,393	98	242,188
		Endosphere	51	54,343,083	1,116,829	56	170,755
Clatskanie, OR	25/52	Soil	52	87,586,266	1,661,751	202	346,257
		Rhizosphere	51	93,804,531	1,745,210	182	357,687
		Endosphere	50	58,547,543	823,103	28	103,709

Average number of genomes represented is calculated within the JGI Genome Portal based on homology of conserved single copy genes^28^.

### Plant transcriptomic sequencing and data processing

Stranded RNASeq libraries were created and quantified by qPCR. Sequencing was performed using an Illumina NovaSeq 6000 instrument. Filtered reads from each library were aligned to the reference genome using HISAT2 version 2.2.0^29^. Strand-specific coverage bigWig files (fwd and rev) were generated using deepTools v3.1^30^. featureCounts^31^ was used to generate the raw gene counts (counts.txt) file using gff3 annotations. Only primary hits assigned to the reverse strand were included in the raw gene counts. Raw gene counts were used to evaluate the level of correlation between biological replicates using Pearson’s correlation. In the heatmap view, the libraries were ordered as groups of replicates. The cells containing the correlations between replicates have a purple (or white) border around them. FPKM and TPM normalized gene counts are also provided. The previous analyses were conducted to provide an assessment of the quality of the data.

### Metabolomic analyses and data processing

For analysis of polar metabolites with LC-MS, normal phase chromatography was performed using an Agilent 1290 UHPLC stack in line with a Q Exactive Orbitrap MS (Thermo Scientific, San Jose, CA). Spectra was collected in centroid mode from *m/z* 70–1050 at 70k resolution in both positive and negative ionization mode, and MS/MS fragmentation data acquired using 10, 20, and 40 eV collision energies (stepped then averaged) at 17.5k resolution. The LC was equipped with a HILIC column (InfinityLab Poroshell 120 HILIC-Z, 2.1 × 150 mm, 2.7 µm, Agilent, #683775-924) held at 40 °C, with mobile phase solvents running at a flow rate of 0.45 mL/min. For each sample, 2 µL were injected onto the column that was first equilibrated with 100% buffer B (99.8% 95:5 v/v acetonitrile:H_2_O and 0.2% acetic acid, w/ 5 mM ammonium acetate) for 1 minute, followed by a 10 minutes linear gradient to dilute buffer B down to 89% with buffer A (99.8% H_2_O and 0.2% acetic acid, w/ 5 mM ammonium acetate and 5 µM methylene-di-phosphonic acid), then down to 70% B over another 4.75 minutes, and finally down to 20% B over 0.5 minutes, isocratic elution for 2.25 minutes. This was immediately followed by column re-equilibration by returning to 100% B over 0.1 minute and isocratic elution for 3.9 minutes. Source settings of the mass spectrometer included ion transfer tube temperature of 400 °C, sheath gas flow rate of 55 (au), auxiliary gas flow of 20 (au), sweep gas flow of 2 (au), and spray voltage of 3 kV.

For analysis of non-polar metabolites, reverse phase chromatography was performed using an Agilent 1290 UHPLC stack in line with an ID-X Orbitrap Tribrid MS (Thermo Scientific, San Jose, CA). Spectra was collected in centroid mode from *m/z* 80–1200 at 60k resolution in both positive and negative ionization mode, with MS/MS fragmentation data acquired using 10, 20, and 40 eV collision energies (stepped and averaged) at 30k resolution. The LC was equipped with a C18 column (Agilent ZORBAX Eclipse Plus C18, Rapid Resolution HD, 2.1 × 50 mm, 1.8 μm) held at 60 °C with mobile phase solvents running at a flow rate of 0.4 mL/min. For each sample, 2 µL were injected onto the column that was first equilibrated with 100% buffer A (100% H_2_O w/ 0.1% formic acid) for 1 minute, followed by a linear gradient to dilute A down to 0% with buffer B (100% acetonitrile with 0.1% formic acid) over 7 minutes, and isocratic elution for 1.5 minutes. This was immediately followed by column re-equilibration by returning to 100% A over 1 minute and isocratic elution for 1 minute. Mass spectrometer source settings included a sheath gas flow rate of 50 (au), auxiliary gas flow of 10 (au), sweep gas flow of 1 (au), spray voltage of 3.5 kV for positive and 2.5 kV for negative ionization, ion transfer tube temperature of 350 °C and vaporizer temperature of 350 °C.

Samples consisted of 3 biological replicates each and 3 extraction controls, with sample injection order randomized and an injection blank of 2 µL of 100% methanol run between each sample, with the blank replaced by an injection of internal standard mix every 3rd sample as well as QC mix every 15 samples.

## Data Records

Metabolomics data is deposited in the MassIVE data repository, accession number MSV000090886^32^. Metagenomics data and plant transcriptomics are available through the JGI under proposal ID 507130^33^ as well as the National Center for Biotechnology Information (NCBI)^34^. A list of NCBI accession numbers, correlated with the *Populus* host identifier, is summarized in Supplementary Table 1 for metagenomics data and Supplementary Table 2 for transcriptomics data. Sample metadata, including sample type, collection methods, time and location information, soil chemical information, and links to the associated metagenomics, transcriptomics, and metabolomics data are available through the National Microbiome Data Collaborative^35^. The soil measurement data is publicly available through the DOE Office of Scientific and Technical Information^36^.

## Technical Validation

Technical validation of the four datasets was performed using established best practices specific for each data type. For metagenomics data, sequencing reads were quality controlled for length (>50), quality (>Q30) and any adapter contamination removed. This parsed read set was then assembled using metaSPAdes assembler version 3.15.2^26^. Further annotation and data processing was performed using the DOE-JGI Metagenome Annotation Pipeline (v.5.0)^28^. For root endosphere metagenomic samples, plant background contamination was assessed across the dataset using the phylogenetic profiler tools within IMG where all genes identified in each metagenome were compared against all other known genomes in the IMG database (Fig. 2). On average, genes with best match to the phylum Streptophyta (including all vascular plants) comprised 23.2% of the endosphere metagenomes but had wide variability (SD = +/− 22.9%). Other microbial phyla identified as present in abundance comported well with those expected in *Populus* root endosphere environments based on past culture and rRNA metabarcoding based assessments^22,37^.

For plant root transcriptomics, raw fastq file reads were filtered and trimmed using the JGI quality control pipeline resulting in the filtered fastq file (*.filter-RNA.gz files). Using BBDuk^38^, raw reads were evaluated for artifact sequence by kmer matching (kmer = 25), allowing 1 mismatch and detected artifact was trimmed from the 3′ end of the reads. RNA spike-in reads, PhiX reads and reads containing any Ns were removed. Quality trimming was performed using the phred trimming method set at Q6. Finally, following trimming, reads under the length threshold were removed (minimum length 25 bases or 1/3 of the original read length, whichever is longer). The average rRNA contamination in this dataset was 0.99% of raw reads. A little over 95% of the raw reads mapped to a genome. There were between 14–151 M genome mapped reads per library.

For targeted metabolomics, internal standards (ISTDs) were added to every sample. These are heavy isotope labeled samples and are used for quality control. The labeled peaks can be compared to corresponding unlabeled peaks in the data set to determine peak accuracy. In the targeted identifications file, column “U” with the heading “retention time of max intensity MS1” is the measured retention time within this experiment. Likewise, column “AH” with the heading “Theoretical retention time (peak)”, contains the expected retention time based on the retention time of running an authentic standard and correcting it for shifts over time.

For environmental metadata, there were several types of analyses performed including soil chemistry, nitrification/denitrification assays, soil particle size analysis, and soil moisture determination. For soil chemistry, an instrument calibration was performed to determine uncertainty budgets for each element, and for pH. For nitrification/denitrification assays, blanks were included in every group of assays and 10% of the soil samples were analyzed in duplicate to assess any potential instrument drift and bias. For the denitrification assays, the resultant N_2_O concentrations were analyzed with a Shimadzu GC-2014 calibrated with analytical standards bounding the observed concentrations. For the nitrification assays, nitrate was determined using colorimetric spectrophotometer methods with a Seal Analytical analyzer. For particle size, the hygrometer uncertainty was measured at 0.5 Ru units. For soil moisture measurements, duplicate measurements were taken, and the final data product was averaged among them to allow for a more accurate result. Uncertainty calculations were done according to standard operating procedures.

## Usage Notes

Programmatic access to the Bio-Scales study metadata at NMDC can be achieved by using the NMDC study identifier for the Bio-Scales project: nmdc:sty-11-r2h77870

The cURL request to realize the above is as follows:

curl -X ‘GET’ \

‘https://api.microbiomedata.org/data_objects/study/nmdc%3Asty-11-r2h77870’\

-H ‘accept: application/json’

The study record includes GNPS, MassIVE and JGI Genome Portal identifiers. The NMDC uses Compact Uniform Resource Identifiers (CURIES) to store identifiers. GNPS, MassIVE, and JGI Genome Portal identifiers can be resolved by using https://bioregistry.io/. For example, a gnps_task_identifiers value of gnps.task:4b848c342a4f4abc871bdf8a09a60807 can be resolved with https://bioregistry.io/gnps.task:4b848c342a4f4abc871bdf8a09a60807.

Similarly, sample metadata can be retrieved programmatically as shown below.

The following is a query to retrieve all metadata about sample nmdc:bsm-11-6zd5nb38 from NMDC

curl -X ‘GET’ \

‘https://api.microbiomedata.org/biosamples/nmdc%3Absm-11-6zd5nb38’ \

-H ‘accept: application/json’

### Supplementary information

Supplementary Table 1

Supplementary Table 2

## Data Availability

The software used for processing the data is described in the methods. A custom Python code^39^, manual curation, and MetAtlas^40^ were used for analysis of LC-MS data. Metagenomic analyses used the DOE-JGI Metagenome Annotation Pipeline (v.5.0)^28^.
